# Pumpkin Waste as Livestock Feed: Impact on Nutrition and Animal Health and on Quality of Meat, Milk, and Egg

**DOI:** 10.3390/ani9100769

**Published:** 2019-10-08

**Authors:** Laura Patricia Valdez-Arjona, Mónica Ramírez-Mella

**Affiliations:** 1 Agricultural Bioprospection and Sustainability for the Tropics Program, Postgraduate College Campus Campeche, Campeche 24450, Mexico; valdez.laura@colpos.mx; 2 National Council of Science and Technology—Postgraduate College Campus Campeche, Campeche 24450, Mexico

**Keywords:** *Cucurbita* sp., agricultural by-products, plant residues, animal feed

## Abstract

**Simple Summary:**

Pumpkin waste are part of the millions of tons of vegetable residues produced yearly that could be used in livestock feeding. Their value not only relies in its nutritional content as its bioactive compounds could modify meat, milk, and egg composition which are of uttermost value for human nutrition. Furthermore, pumpkin waste, which cannot be used in human consumption, may contribute to diminish human-livestock competition for cropland. In this review, we describe the potential of pumpkin waste as animal feedstock as a strategy for more sustainable livestock production while making emphasis on the importance of food from animal origin in human health.

**Abstract:**

Meat, milk, and egg contribute positively to the nutrition and health of humans; however, livestock requires a large number of resources, including land for fodder and grains. Worldwide millions of tons of vegetable waste are produced without any further processing, causing pollution and health risks. Properly managed vegetable waste could provide a source of feed for livestock, thus reducing feeding costs. In this regard, pumpkin waste (*Cucurbita* sp.) is an alternative. Research on pumpkin waste on animal nutrition is scarce, however, it has potential as animal feed not only for its nutritional value but also for its antioxidants, pigments, and polysaccharides content that could enhance quality of meat, milk, and egg, as well animal health. In this review, we describe the environmental impact of livestock as a result of greater demand for food of animal origin, including the importance of the consumption of animal foods in human nutrition and health. Moreover, we emphasize the potential of plant residues and, particularly, on the characteristics of pumpkins and how their use as feedstuff for livestock could improve productivity and modify the composition of meat, milk, and egg.

## 1. Introduction

As a result of the worldwide increase in the human population [1], the demand for meat, milk, and eggs will increase in subsequent years, therefore, there will be more demand for fodder to feed livestock [2]. This will increase the production of greenhouse gases originated by livestock [3]. Reducing meat consumption could help reduce greenhouse gas emissions [4]; however, the consumption of food of animal origin positively impacts not only health but also the human state of mind [5].

Currently, the aim is toward more sustainable livestock based on the efficient use of available food resources, thus reducing competition with humans for farmland and grains for animal feed [6]. Vegetable residues contain various bioactive compounds, such as vitamins, unsaturated fatty acids, and phytochemicals [7], which can benefit the health and productivity of animals. Among these plant residues are pumpkins. The composition of pumpkins varies depending on the species and part of the plant [8]. In general, the fruit is a source of carbohydrates, vitamins, minerals, pigments, phenolic acids, and flavonols [9,10]; while the seeds provide protein and fatty acids [8,11]. In addition, pumpkins have been described to have medicinal and pharmacological properties [9,11,12]. Research on the use of pumpkins in animal feed is scarce and its benefits in the productivity and quality of meat, milk, or egg are attributed to its protein and fat content in the case of seeds, and carbohydrates, minerals, and vitamins in the case of the fruit [13].

In this review, we summarize the importance of using ingredients not suitable for human consumption (as plant residues) in animal feed. With these ingredients, food of animal origin can be produced. Emphasis is placed on describing the characteristics of pumpkins and how their use in the diet of livestock may contribute to improve the productivity and composition of meat, milk, and eggs. Additionally, the importance of the consumption of animal foods in human nutrition and health are discussed.

## 2. Human Population and Food Demand

The world population was 7600 million by 2017 and it is estimated that by 2050 it will be 9700 million, therefore increasing the demand for food. Under this scenario, it is expected that by 2050, the consumption of meat and eggs will increase by 73% and of milk by 59%, compared to 2010 [1]. The demand for products of animal origin increases the demand for the forage needed to feed livestock. In 2017, 1.6 billion tons of fodder was used globally to produce meat, eggs, and milk, and the demand will increase as livestock production intensifies [2]. Until 2015, agricultural expansion was 4900 million hectares, and from 2010 to 2015, 3.3 million hectares were deforested each year. In fact, of the total land allocated to agriculture, the third part is used for livestock activities. Therefore, global food security is at risk due to increasing pressure on natural resources threatening the sustainability of food systems in general and, if this trend continues, the limits of planet Earth could be exceeded [14].

Furthermore, there has been a change in the eating habits of human populations due to several factors among which we can emphasize the income of the population, urbanization, and globalization of the markets, causing traditional diets to be replaced by diets with higher content of sugars, fats, and meat. It is clear that the demand for protein of animal origin increases when the population has higher income and, as the demand for protein of animal origin increases, the demand for protein from legumes decreases [4]. In the other hand, with the growth of the human population, the demand for food of animal origin increases and, as a consequence, the production of greenhouse gases caused by livestock and other related activities, such as agriculture, industry, and transport, increases. Livestock contributes with 14.5% of greenhouse gas emissions of anthropogenic origin, estimated at 7.1 gigatons of CO2-eq per year, of which beef and milk cattle contribute 41% and 20%, respectively, while pigs and poultry (for meat and eggs) contribute 9% and 8%, respectively. Fodder production, enteric fermentation of ruminants, and manure account for 45%, 39%, and 10% of total greenhouse gas emissions from livestock, respectively. The remaining emissions corresponding to 6% are attributed to the processing of products of animal origin and transport for its distribution [3].

## 3. Food of Animal Origin and Its Impact on Human Health

Greenhouse gas emissions are directly related to the type of diet of the population. This means that food can impact the environment in a different way even when they are similar with respect to their nutritional value or effect on health. For example, Mediterranean, Pescetarian, and Vegetarian diets reduce greenhouse gas emissions by 30%, 45%, and 55%, respectively. However, diets with low environmental impact do not always contribute to health. Diets with a high content of sugar, starch, and fat have a low environmental impact but can contribute to the development of diseases such as type II diabetes [4], whereas an adequate diet that includes meat positively impacts the mental and cognitive state, although meat as itself is highly demanding of resources (water, land, grains) and generates waste (greenhouse gases, manure) [5]. In the last decades, an interest in developing functional foods has emerged, including those of animal origin, which contain diverse elements (such as proteins or specific fatty acids) capable of preventing chronic diseases or cancer [15].

### 3.1. Meat

Meat is one of the protein sources with the highest biological value and best digestibility. According to the Protein Digestibility-Correct Amino Acid Score (PDCAAS), meat has a score of 0.92 (beans or lentils, legumes considered as one of the most important sources of vegetable protein, oscillate between 0.57 and 0.71). Meat provides all the essential amino acids and it is a source of the B-complex-vitamins and minerals such as zinc, selenium, phosphorus, and iron [16]. In addition to its nutritional contribution, meat consumption was a determining event in human evolution. Meat is a source of tryptophan which is a precursor to nicotinamide, also known as vitamin B3; its deficiency has effects on serotonin and, consequently, on social behavior and cognition [5]. Nicotinamide is directly related to neuronal differentiation and neurodegeneration in the central nervous system, and its deficiency seems to be implicated in diseases such as Alzheimer’s, Parkinson’s, and Huntington’s, as well as dementia [17].

### 3.2. Milk

Cow’s milk is one of the food products with the greatest nutritional contribution [18]. A single glass of milk can cover an important part of the daily requirement of some vitamins and minerals. Additionally, milk is a source of protein of high biological value, whose score according to PDCAAS is one of the highest, between 0.95 and 1 [19], even higher than meat [16]. Casein is the most important protein in milk, constituting more than 80% of the total protein; the rest is made up of lactoglobulins, immunoglobulins, and serum albumin. Milk also contains lactose (the main carbohydrate in milk) and fat. Milk fat is made up of more than 95% triglycerides, of which more than 60% are saturated fatty acids and about 30% monounsaturated fatty acids, while the contribution of polyunsaturated fatty acids is low due to ruminal biohydrogenation. Additionally, milk also contains cholesterol [18].

Due to the high content of saturated fat in milk, its consumption has been related to the development of heart disease, overweight, and obesity; however, stearic acid (one of the main saturated fatty acids in milk) is not considered to be atherogenic and does not increase the concentration of blood cholesterol, while lauric acid (another saturated fatty acid present in milk) can eliminate *Helicobacter pylori*, a bacterium related to gastric problems and stomach cancer [20]. Moreover, it has been shown that there are components in the milk fat globules with anticancer, anticholesterol, antidepressant, bactericidal, and antioxidant properties, among others. Several of these compounds correspond to proteins found in the membrane of fat globules, for example, mucin 1, butyrophilin, xanthine oxidase, caveolin, BRCA1, and fatty acid-binding protein (FABP). In fact, FABP has the ability to inhibit breast cancer cell lines at very low concentrations [15]. Additionally, ruminant milk contains conjugated linoleic acid, a fatty acid that is formed from ruminal biohydrogenation. Some conjugated linoleic acid isomers have been of interest due to their properties to prevent cancer, decrease the formation of atheromas, improve the immune response, and change body composition [21].

### 3.3. Egg

Even before animal domestication, humans consumed eggs from hens and other birds, thus obtaining from this food lipids, amino acids, vitamins, minerals, and bioactive compounds. Egg is also a cheap food with high nutritional value that contributes to cover the nutritional requirements of several human societies [22,23]. Nonetheless, half a century ago it was demonized due to its cholesterol content. 50 g of egg contains 186 mg of cholesterol which is vastly higher than that from 100 g of meat or milk with 67 mg and 10 mg, respectively, thus exceeding by 50% the daily intake. For this, it was recommended not eating more than three yolks per week with the aim of reducing plasmatic cholesterol in the population [22]. It is difficult to modify egg cholesterol content, however, its lipid profile can be modified through hen feeding by reducing the content in fatty acid omega-6 whereas increasing fatty acid omega-3 [24]. The use of polyunsaturated oils such as linseed in rations for hens increases eicosapentaenoic acid content whereas soy increases docosahexaenoic acid in egg yolk [25]. Both eicospentanoic acid (20:5) and docosahexanoic acid (22:6) are omega-3 long-chain fatty acids whose consumption is associated with a lower incidence of coronary disorders, diminished inflammatory processes as well as brain and retina development [26]. Furthermore, eggs are source of antioxidants, like vitamin E, carotenoids, and selenium [27], and it is an important source of proteins. Ovoalbumin is the main protein from albumin which also contains ovotransferrin, ovomucin, lysozyme, avidin, cystatin, ovoinhibitor y ovomacroglobulin. Egg proteins possess diverse biological functions that benefit human health due to its antimicrobial, antiviral, antitumoral, antiadhesive, antioxidant, and immunomodulatory activities [28]. Thus, egg can be considered a “functional” food [27] with potential to prevent disease in humans [28].

## 4. Plant Residues in Animal Feed

Due to population growth, more food production is required to improve food security [29], so those involved in the nutrition of animals intended for human consumption are challenged to optimize food efficiency, obtaining more meat, milk, and egg but with less feed for the animals’ diet [30]. There is also a need to restrain and reduce the environmental impact of livestock, using less land, water, energy, and fertilizers [31] and reducing greenhouse gas emissions [7]. A strategy to achieve these objectives is to use the waste generated by agriculture, such as plant residues or by-products of the food industry [32] to feed animals intended for human consumption [33]. Derived from the demand for food of animal origin for human consumption, production systems require a shift toward more sustainable livestock, based on the efficient use of available food resources, reducing waste, and using new food sources, particularly those that are not destined for human consumption [6]. Under this context, plant residues are key to achieve this objective. In fact, the use of plant waste for animal feed is not new, as it is a common practice in rural areas [34].

The incorporation of plant waste into animal feed could reduce production costs [6]. This is particularly important as feeding represents one of the most expensive items of livestock systems. However, it is necessary to emphasize that the use of plant residues in animal feeding does not always imply a reduction in production costs since factors such as the processing itself can increase the price of the diet. For example, due to their high moisture content, agricultural residues require drying to be preserved for long periods of time. The environmental benefit must be taken into account by decreasing the amount of waste that reaches the landfills [35] and a lower proliferation of insects, which find plant waste an ideal place for reproduction [36]. Additionally, the social benefit must be taken into account by reducing the use of grains in animal feed.

Currently, there is no estimate on the amount of agricultural waste that is generated, mainly because of the absence of records and the fact that the harvest seasons are different every year [31] and that waste has no economic value since it does not have a place in any market [37]. However, the FAO notes that one-third of all food produced is wasted [14,37], being the highest waste rate from fruit and vegetables [38], and more than 40% of the losses of foods are generated in the post-harvest and processing stages [39]. China is one of the countries that generate more plant waste, producing about 32 million tons annually, while the United States of America produces 15 million tons, which at best, are used in compost production or simply are deposited in landfills or dumpsites [6]. The use of plant waste as soil fertilizer, as raw material to generate biofuels [37], and as feed for small farms has also been reported [29]. In this way, plant waste can continue to be part of the human food chain [37] as livestock have the ability to recycle nutrients from this type of waste, which are no longer suitable for human consumption, by converting them in nutritious foods. Thus, using plant waste as animal feed avoids competition between foods and fodder [7] as well as contributing to the efficient use of local agricultural resources [31].

A major limitation of plant waste is its high moisture content that makes handling difficult and favors its rapid decomposition. Therefore, it is necessary to use some form of conservation of this waste [32], without diminishing its quality [35]. In addition, it should be considered that plant residues are not always produced constantly throughout the year, thus most of the production is available only during certain months of the year [40]. Consequently, plant residues could be a feed alternative during the dry season when other resources are depleted [41]. Silages are an option that could be used to conserve plant residues for a longer time [42,43] as there are examples of their application in various fruits and vegetables, such as cassava, beets, carrots, broccoli, squash, various citrus fruits, banana, and pineapple [44,45,46]. Due to its high moisture content, plant residues cannot be silage alone, so they are mixed with straw or stubble [6]; in this way, the mixture of vegetable/straw residue has a dry matter level between 25% and 40% for its correct fermentation process [47]. Drying and subsequent transformation into flour can also be a viable storage alternative [48]. In order to achieve this, the use of solar dryers can be considered or simply through direct drying under the sun [6].

Plant residues contain various bioactive compounds, such as vitamins, unsaturated fatty acids, and phytochemicals, which can benefit the health and productivity of animals [7]. Several assessments have shown that plant residues can be used as part of the livestock diet, replacing part of other ingredients, without affecting weight gain, milk production, or egg-laying [34,49,50]. In general, the peel, pulp, and seeds of vegetables are a source of polyphenols, which have anticancer, antimicrobial, antioxidant, and immune system stimulant properties. Essential oils are obtained from the peel of some citrus fruits that can prolong the shelf life of food. Plant residues can also contain antioxidants that act by eliminating free radicals and preventing the formation of peroxides. Additionally, plant residues can be a source of various enzymes such as bromelain (pineapple), papain, amylase, laccase, and manganese peroxidase, which have various biological functions and biotechnological applications [6].

## 5. World Overview of Pumpkin Production

**Figure 1:**
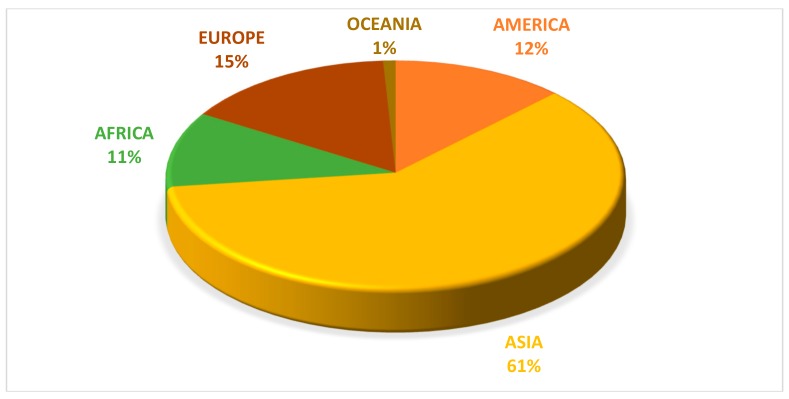
Distribution of pumpkin production in the world, per continent (2017). Source: FAO [51].

**Figure 2:**
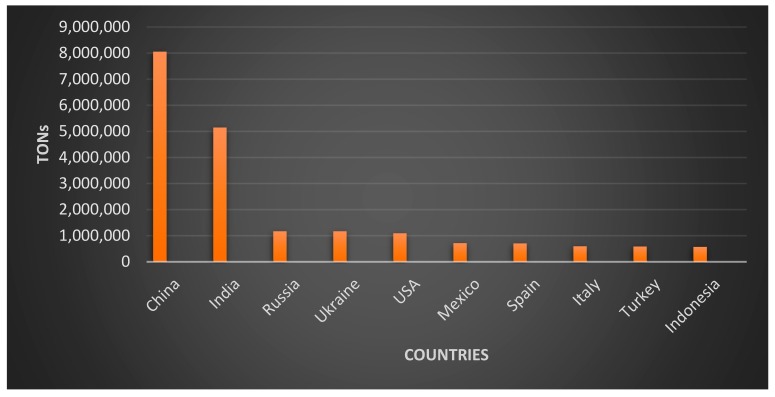
Main pumpkin producing countries in the world (2017). Source: FAO [51].

According to FAO [51], global pumpkin production from 1994 to 2017 was more than 27 million tons. Although its production was distributed across all continents, Asia was the world’s leading producer of pumpkins (Figure 1). In 2017, China and India produced 7,996,362 and 5,142,812 tons respectively, contributing in 48% to the global production of pumpkins followed by Russia, Ukraine, the United States of America, and Mexico (Figure 2).

## 6. The Genus Cucurbita

Cucurbitaceae play a fundamental role in the economy and culture of various societies. Many of the species in this family were the first plants domesticated by humans, being used as food or medicine [52]. Although currently the highest production is found in Asian countries [51], the genus *Cucurbita* is native to America. Before the arrival of the Spaniards in 1492, several species of pumpkins were pillars of pre-Hispanic agriculture and were traditionally cultivated together with corn (*Zea mays*) and beans (*Phaseolus vulgaris*). In this way, the corn served as a support for the beans and provided shade for the pumpkin; meanwhile, beans fixed nitrogen in the soil, while pumpkin prevented loss of soil moisture and weed growth [53]. In Mexico, this sowing system is known as “milpa” [54]. After the arrival of the Spanish conquerors in American lands, the existence of pumpkins in other continents was reported and the evidence was published between 1503 and 1508 (only 11 and 16 years after the arrival of Christopher Columbus to the New World) as the first image of a *Cucurbita* outside of America in the book Grandes Heures d’Anne de Bretagne, in an illustration made by Jean Bourdichon in Touraine, France and corresponding to a *Cucurbita pepo*. Subsp. texana. However, the arrival of domesticated species of *Cucurbita* to Europe took place a decade later [55] where they continued to be harvested.

The Cucurbitaceae family is one of the important ones among vascular plants; it includes 118 genera and 825 species. Mexico is the most important center of diversity where 34 genera (five of them endemic) and 142 species are found [52]. Among the Cucurbitaceae family, the genus *Cucurbita* is found, in which 9 species are described (Table 1) [56]. From these, *Cucurbita argyrosperma*, *Cucurbita maxima*, *Cucurbita moschata,* and *Cucurbita pepo* are harvested and have importance in terms of agricultural production [53], while *Cucurbita okeechobeensis*, a wild species, is practically extinct. The decrease and extinction of wild species of *Cucurbita* is directly related to the decrease of the megafauna responsible for the seed dispersal and the loss of habitat. The wild and domesticated species of *Cucurbita* present notable differences. Most domesticated subspecies show a greater variety of colors, shapes, and sizes than wild species. In addition, in the cultivated species, domestication has favored germination to be more uniform, and the size of fruits and seeds has increased; however, resistance to diseases and pests has been reduced [57].

The different species of *Cucurbita* are cultivated for their seeds and fruits, which are mainly used as food. In addition to the fruit, the seeds are probably one of the most important objectives for which some pumpkin species are harvested. Once they are collected, cleaned, and baked, the seeds can be consumed directly as a “snack”. They are also ground to make a kind of pasta, with which various dishes are prepared [53]. In some cases, stems, leaves, and flowers are also consumed. Furthermore, the uses that have been given to pumpkins are very diverse, for example, it has been reported that the peels have been used as containers, mainly by semi-nomadic people before the pre-ceramic era, or, due to their saponin content, the pulp is used to make soaps [57].

## 7. Nutritional and Phytochemical Composition of Pumpkins

The nutritional composition of pumpkins is variable and depends on several factors, among which are the growing conditions, the species, and the part of the plant or fruit [13]. In a study by Kim et al. [8], in which three species of pumpkin (*Cucurbita pepo*, *Cucurbita moschata,* and *Cucurbita maxima*) were evaluated, they reported that *Cucurbita maxima* contains significantly more carbohydrates, fat, and fiber, while *Cucurbita pepo* and *Cucurbita moschata* have higher protein content. The seeds are the ones that concentrate the majority of carbohydrates, protein, and fat with the exception of *Cucurbita maxima* where the peel and pulp concentrate a higher amount of carbohydrates compared to the seeds (Table 2). Dorantes et al. [58] reported 8% protein, 3% fat, and 49% neutral detergent fiber in the dry peel and pulp residue of *Cucurbita argyrosperma*. In this report, the low protein and fat content is due to the fact that the pumpkin did not contain the seeds, in which these nutrients are concentrated. On the other hand, Zhang et al. [59] reported that the dried pulp of *Cucurbita moschata* contains 44% sugars and 9% pectin.

Additionally, pumpkins contain various minerals such as copper, manganese, potassium, calcium, iron, magnesium, zinc, and selenium, and vitamins A, E, C, and of the B-complex [8,13,58,60]. According to Dorantes et al. [58], the dry peel and pulp residue of *Cucurbita argyrosperma* contains 1.12% calcium, 0.18% phosphorus, and 0.18 mg of magnesium. On the other hand, Elinge et al. [61] reported that *Cucurbita pepo* seeds contain calcium, magnesium, sodium, phosphorus, iron, zinc, manganese, and cobalt (Table 3). Regarding the vitamin content, it is well known that pumpkins are an important source of carotenoids [8,12,60,62]; as the yellow or orange color of the pulp indicates high carotene content [63]. Azevedo et al. [60] identified several carotenoids present in the pulp of several species and varieties of pumpkins, among which are neoxanthin, violaxanthin, lutein, zeaxanthin, α-cryptoxanthin, α-carotene, β-carotene, and ζ-carotene. It was determined that the pulp of *Cucurbita pepo* has the lowest concentration of carotenoids, *Cucurbita moschata* has the highest concentration of α-carotene (23.8 to 26.8 µg/g) and β-carotene (56.7 to 66.7 µg/g), while the hybrid between *Cucurbita maximum* and *Cucurbita moschata* has a higher content of lutein (56.6 µg/g) and neoxanthin (14.4 µg/g). It should be noted that in the pulp of *Cucurbita pepo* and *Cucurbita maxima*, α-carotene was not detected, whereas, in *Cucurbita moschata,* violaxanthin was not detected; these are similar to those reported by Kim et al. [8] who mentions that the pulp of *Cucurbita pepo* has the lowest content of β-carotene (1.48 mg/kg) and β-cryptoxanthin (not detectable) compared to *Cucurbita moschata* (5.70 mg/kg and undetectable, respectively) and *Cucurbita maxima* (17.04 mg/kg and 0.65 mg/kg, respectively), with the content of β-carotene being higher in the pulp of *Cucurbita maxima*. In the three species studied by Kim et al. [8], the highest content of β-carotene was found in the peel, being considerably higher in *Cucurbita maxima* (123.19 mg/kg) compared to *Cucurbita moschata* (68.30 mg/kg) and *Cucurbita pepo* (39.48 mg/kg). On the other hand, Badr et al. [12] mention that the highest content of β-carotene is present in the pulp (39.34 µg/g), followed by the peel (7.51 µg/g), and finally the seed (0.78 µg/g) of *Cucurbita pepo*. These data are interesting considering that in many cases, such as it happens in Mexico with *Cucurbita argyrosperma*, the fruit of the pumpkins is discarded [13]. To our knowledge, there are no reports of *C. argyrosperma* content of vitamins and antioxidants contents. Thus, it remains to be determined in order to consider its pulp and peel value.

The content of the phenolic acids and flavonols in pumpkins has also been described. Recently Kulczyński and Gramza-Michałowska [10] identified nine phenolic acids in the pulp of different varieties of two pumpkin species (*Cucurbita pepo* and *Cucurbita moschata*), such as gallic acid, protocatechuic acid, 4-hydrxybenzoic acid, vanilic acid, chlorogenic acid, caffeic acid, p-coumaric acid, feluric acid, and sinapic acid, as well as six flavonols, including rutin, kaempferol, isoquercetin, astragalin, myricetin, and quercetin, whose concentrations depend on both the species and the variety of pumpkin. According to Zdunić et al. [64], the amount of total phenolic compounds is around 906 ug of GAE/g of fresh fruit of *Cucurbita moschata*. Phenolic compounds have also been described in pumpkin seed oil (24.7–50.9 mg of GAE/kg of oil), such as tyrosol, vanillic acid, vanillin, luteolin, and sinaptic acid [65]. In addition, seeds contain squalene (583–747 mg/100 g), a triterpene that is precursor of steroidal hormones, cholesterol, and vitamin D. Furthermore, pumpkin seeds have sterols, of which the most abundant are Δ^7.22.2^5-stigmastatrienol and Δ^7^ sterol spinasterol, with concentrations between 18.8 and 35.1 g/100 g and between 18.2 and 23.3 g/100 g of total sterols, respectively [9].

In addition to the previously mentioned elements, it has been documented that pumpkins may contain some anti-nutritional compounds, including phytates, oxalates, nitrates, and hydrocyanic acid present in the seeds without serious health problems having been reported when consumed by the animals [13]. This is probably because the concentration in which these compounds are found is below the limit at which they could cause a health problem [61]. Additionally, it has also been reported that pumpkins contain cucurbitacins. Cucurbitacins are a group of tetracyclic triterpenes known for their bitter taste and toxicity, with a cucurbit skeleton ([19- (10 → 9β) -abeo-l0α-lanost-5-ene] also known as 9β-methyl-19- nor lanosta-5-ene) which is highly unsaturated [66] and contain numerous keto-, hydroxy-, and acetoxy groups, characteristics that make cucurbitacins different from other tetracyclic triterpenes. Cucurbitacins can be found in a free form, as aglycone, or as a monoglucosylated compound, having a unit of D-glucose or L-rhamnose. Cucurbitacins are crystalline substances; most of them are soluble in petroleum ether, chloroform, benzene, ethyl acetate, methanol, and ethanol and are slightly soluble in water. They have a UV light absorption between 228 and 234 nm [67]. Different aglycone cucurbitacins have been isolated, which have been divided into twelve categories and have been identified from letter A to T [67,68]. The most common cucurbitacins of the Cucurbitaceae family are cucurbitacin B and D [67].

The first cucurbitacin was isolated in 1831 and received the name of α-elaterin. Cucurbitacins have been identified both in the Cucurbitaceae family and in other plant families. The concentration of cucurbitacins varies among different tissues; higher concentration in fruits and roots of mature plants and lower in leaves and stems. Seeds generally contain low concentrations of cucurbitacins. Probably, the main drawback of cucurbitacins is their toxicity. The toxic potential of cucurbitacins was first described in 1932 but received special attention in 1981 when a highly toxic and bitter compound, cucurbitacin E, was identified in cans of *Cucurbita pepo*, in California, USA [67]. Studies in mice indicate that the most toxic cucurbitacin has a lethal dose of 5 mg/kg of body weight while the least toxic is 650 mg/kg of body weight; however, it seems that the toxicity depends on the species since some animals such as coyotes and porcupines show tolerance to the consumption of the pulp and seeds of wild pumpkins, while wild boars ingest the roots of *Cucurbita foetidissima* and *Cucurbita digitata* [53].

## 8. Impact on Livestock Nutrition and Productivity

There is evidence that pumpkins are used traditionally and on a small scale in the feeding of several species of domestic animals such as ruminants [69] and equines [53,70]. Research on the use of pumpkins in animal feeding and its productivity benefits are attributed to its protein and fat content in the case of seeds, and carbohydrates, minerals, and vitamins in the case of the fruit [13].

Regarding the fruit, Dorantes et al. [58] mention that the dry residue of *Cucurbita argyrosperma* (peel and pulp only) has low protein content (<9%) but contains almost 50% of neutral detergent fiber and 40% of acid detergent fiber, which makes it suitable for the formulation of diets for dairy cattle and rabbits. On the other hand, Crosby et al. [71] mention that the ruminal digestibility of dry matter increases 21% by replacing up to 30% of corn stubble with dry residue of *Cucurbita argyrosperma*; however, the digestibility of neutral detergent fiber decreases 7%, which is attributed to the degradation of nonfibrous carbohydrates such as sugars, which are rapidly fermented. Possibly, the sugar content is one of the reasons why the incorporation of pumpkin improves the palatability of the diet [72]. Pumpkin fruits can also be successfully silage, provided they are mixed with other ingredients with lower moisture content, for example, 20% beet pulp. In this case, beets improve silage characteristics by providing easily fermentable carbohydrates [72,73].

To date, studies on the use of pumpkins in ruminant feeding are scarce. In cattle, Halik et al. [74] reported an increase of about 6 kg/d of milk by replacing 17% of corn silage with *Cucurbita maxima* silage (20.7 kg/d vs. 26.5 kg/d, respectively), while in buffalos there was no change in weight gain by including up to 14% of *Cucurbita pepo* silage [75]. Research on sheep has focused on the use of pumpkin seed. In this regard, Klir et al. [76] did not report changes in milk production or its composition by adding 16% of pumpkin seed flour in the diet of dairy goats, completely replacing soybean meal. On the other hand, Antunovic et al. [77] found that replacing soybean meal with up to 15% of pumpkin seed meal in the sheep diet does not alter the color of the meat or the carcass yield. In the latter two studies, the species of pumpkin used are not mentioned.

The largest number of studies regarding the use of pumpkin seeds as animal feed has been conducted in broiler chickens in which an increase in weight gain and carcass yield was observed. In this regard, Martínez et al. [78] reported greater weight, greater breast yield, and lower abdominal fat when 6% of *Cucurbita moschata* seed flour was included in the diet, while Zinabu et al. [79] improved weight gain with only 1% of *Cucurbita maxima* seed flour included in the chicken diet. Hajati et al. [80] showed that an addition of 5 g/kg of pumpkin seed oil to the ration does not affect the productive performance. In laying hens, there were no changes in the laying rate or the quality of the egg using pumpkin seed flour [81,82]. In the case of turkeys, the use of 5% of seeds in their diet improved the fertility of the eggs, reduced embryonic death, and increased hatching rate [83].

In pigs, research on the use of pumpkins as food is scarce; however, the report by Medina et al. [84] indicates that the weight gain of pigs is not affected when up to 30% of the ration is replaced by *Cucurbita pepo* ferment. However, food consumption increases by 75%, which affects food conversion.

Besides its use as food, various medicinal properties have been attributed to pumpkins. In this regard, different compounds have been reported, both in seeds and fruit, with different biological activities: antioxidant, antifungal, antiparasitic, antimicrobial, and anti-inflammatory [11,13]. Therefore, the health and consequently the productivity and welfare of livestock can be improved with the addition of pumpkin to the diet. In vitro assays in birds have reported that *Cucurbita pepo* ethanolic extract is effective against *Histomonas meleagridis*, *Tetratrichomonas gallinarum,* and *Blastocystis* sp. which are protozoans of economic importance in poultry farming; however, it has limited effect in vivo [85]. It has also been shown that pumpkin seed lectins have antibiotic action against *Salmonella typhymurium*, *Salmonella gallinarum*, *Escherichia coli,* and *Pseudomonas*, so their use could decrease the use of antibiotics. Additionally, the whole fruit has action against the New Castle virus [13]. In pigs, the ferment of *Cucurbita pepo* decreases the frequency of diarrhea leading to a decrease in the mortality and morbidity of piglets [84]. In general, the cucurbitacins present in pumpkins have digestive and purgative action due to their bitter compound content [66], which is the reason why pumpkins have most likely been used as antiparasitic agents [11,13,69]. Additionally, Bahramsoltani et al. [86] demonstrated that 20% *Cucurbita moschata* peel extract can be used successfully to treat burns due to the high mucilage content which reduces oxidative stress of burned tissue.

## 9. Effect on the Composition of Meat, Milk, and Egg

An important point to highlight is the use of pumpkins in animal feed to improve the quality of meat, milk, and eggs due to their high content of antioxidants (in the case of pulp and peel) and polyunsaturated fatty acids (present in the seeds). As previously mentioned, the peel and pulp of several pumpkin species contain significant amounts of α-tocopherol and β-carotene [8]; α-tocopherol has been shown to delay lipid oxidation and prevent loss of meat color, prolonging the shelf life of the product [87]. Humans can obtain α-tocopherol and β-carotene through the consumption of meat and milk from animals that received diets with a high content of these antioxidants [88]. In humans, a diet rich in carotenes improves the immune response and reduces the risk of degenerative diseases such as cancer, cardiovascular diseases, atherosclerosis, cataracts, and age-related macular degeneration [89]. Milk, in addition to being a source of nutrients such as fat, lactose, and protein [90], is also a source of several antioxidants including β-carotene [91] and its composition may vary depending on the type of the cow’s diet [20]. In a study carried out with dairy cows, it was observed that the inclusion of 12%–17% of *Cucurbita maxima* silage in the diet significantly increased the content of α-carotene, β-carotene, lutein, and violaxanthin in milk [74].

Because of its carotene content [8], the pulp and peel of pumpkins could be used in the diet of laying hens. The color of the yolk is a characteristic associated with the quality and freshness of the egg according to consumers. The color depends on the accumulation of carotenoids, molecules synthesized by higher plants, algae, bacteria, and fungi, and which birds obtain from the diet, either in a natural form from ingredients that contain them or by a synthetic source. The carotenoids mostly used in the yolk pigmentation are xanthophylls [92]. Β-cryptoxanthin, a type of xanthophyll, has been shown to improve egg yolk coloration. In this regard, Heying et al. [93] improved the color of the egg yolk when they fed laying hens with a corn fortified with β-cryptoxanthin instead of a conventional one. It should be noted that corn is an ingredient widely used in the feeding of poultry due to pigments contribution. With fortified corn, the content of β-cryptoxanthin (8.43 nmol/g vs. 1.55 nmol/g) in egg yolk was also increased. Increased consumption of β-cryptoxanthin has been shown to reduce the risk of developing lung cancer, especially the type induced by smoking [94]. Additionally, lutein content doubled with fortified corn and zeaxanthin content quintupled in the yolk [93]. Hen fed with pumpkin fruit could stabilize the yolk polyunsaturated fatty acids due to higher antioxidant levels in their diet. Stabilizing the fatty acids would extend egg shelf life and avoid stale and rancid odors [27].

It should be considered that drying or silage processing can reduce the antioxidant content, particularly of carotenes and tocopherol [95]. In this regard, Lozicki et al. [73] reported a 33% and 18% reduction in the content of β-carotene and α-tocopherol, respectively, in pumpkin silage/beet pulp (80:20), an effect attributed to a low pH in the silage material, favoring a rapid oxidation rate of unsaturated fatty acids and, therefore, of carotenoids and tocopherols by the action of the enzyme lipoxidase.

Regarding the seeds, Martínez et al. [96] reported that 10% of *Cucurbita maxima* seed meal on the diet of laying hens increases the content of various essential mono and polyunsaturated fatty acids. Furthermore, by increasing the pumpkin meal in the diet, the content of omega-3 fatty acids increased more than double (from 454 mg/100 g in the control group to 1095 mg/100 g of yolk in the treated group with a 10% of pumpkin seed flour added). Moreover, there was a 10% decrease in the cholesterol content in the egg. The previous results could contribute to improve the human diet since the consumption of cholesterol and some lipids is considered unhealthy [13]. Additionally, the use of pumpkin seed meal, up to 100g/kg, in the diet of laying hens does not confer unpleasant flavors to the egg, an effect that has been reported when using meals of other seeds such as flax [82]. Similarly, the dietary inclusion of pumpkin seed could improve the composition of chicken meat. In this regard, Hajati et al. [80] reported a decrease in plasmatic cholesterol and triglycerides of chickens fed with a diet containing 1% of pumpkin seed oil. On the other hand, Antunovic et al. [77] also reported a decrease in blood cholesterol in sheep when they replaced soybean meal with up to 15% of pumpkin seed cake in the diet, without changes in the lipid profile in the semi-membranous muscle. In dairy sheep, pumpkin seed cake increases omega-3 and branched-chain fatty acids, which could have important implications for human health [76].

## 10. Conclusions

This review shows that pumpkins have benefits in the productive performance of livestock when they are part of the diet. Additionally, due to the high content of antioxidants and fatty acids present in the fruit and seeds, respectively, some of the characteristics of meat, milk, or egg are improved, contributing to the human access to a healthier diet. The idea, in any case, is not the harvest of pumpkins for animal feed but to use fruits not suitable for human consumption or the waste generated after harvest or post-processing. Thus, the environmental impact of livestock production is reduced, contributing to the sustainability of animal production systems and to cover the demand for food of animal origin, which impacts the physical and mental health of the human being.

## Figures and Tables

**Table 1:** Pumpkins taxonomic information [56].

Kingdom	Plantae
Division	Tracheophyta
Class	Magnoliopsida
Order	Cucurbitales
Family	Cucurbitaceae
Genus	*Cucurbita* L.
Species	*argyrosperma* *digitata* *ficifolia* *foetidissima* *máxima* *moschata* *okeechobeensis* *palmata* *pepo*

**Table 2:** Chemical composition of the pulp, peel, and seeds (g/kg of raw material) from three pumpkin species [8].

		Species		
Chemical Composition	Part	*Cucurbita pepo*	*Cucurbita moschata*	*Cucurbita maxima*
Carbohydrates	Peel	43.76	96.29	206.78
	Pulp	26.23	43.39	133.53
	Seed	122.20	140.19	129.08
Protein	Peel	9.25	11.30	16.54
	Pulp	2.08	3.05	11.31
	Seed	308.83	298.11	274.85
Fat	Peel	4.71	6.59	8.59
	Pulp	0.55	0.89	4.20
	Seed	439.88	456.76	524.34
Fiber	Peel	12.28	34.28	22.35
	Pulp	3.72	7.41	10.88
	Seed	148.42	108.51	161.54
Humidity	Peel	935.98	871.86	756.79
	Pulp	967.70	942.31	840.43
	Seed	74.06	51.79	27.51

**Table 3:** Mineral content (mg/100 g dry weight) of *Cucurbita pepo* seeds [61].

Calcium	9.78
Magnesium	67.41
Sodium	170.35
Potassium	237.24
Phosphorus	47.68
Iron	3.75
Zinc	14.14
Magnesium	0.06
Cobalt	2.17
